# Changes in Physical Activity and Sedentary Behaviour in Cardiovascular Disease Patients during the COVID-19 Lockdown

**DOI:** 10.3390/ijerph182211929

**Published:** 2021-11-13

**Authors:** Bram M.A. van Bakel, Esmée A. Bakker, Femke de Vries, Dick H.J. Thijssen, Thijs M.H. Eijsvogels

**Affiliations:** 1 Department of Physiology, Radboud Institute for Health Sciences, Radboud University Medical Center, P.O. Box 9101, 6500 HB Nijmegen, The Netherlands; bram.vanbakel@radboudumc.nl (B.M.A.v.B.); Esmee.Bakker@radboudumc.nl (E.A.B.); f.devries111@gmail.com (F.d.V.); Dick.Thijssen@radboudumc.nl (D.H.J.T.); 2 Research Institute for Sports and Exercise Sciences, Liverpool John Moores University, Liverpool L3 5UX, UK

**Keywords:** COVID-19, lockdown, cardiovascular disease, physical activity, sedentary behaviour

## Abstract

The COVID-19 lockdown has been associated with physical inactivity. We prospectively evaluated changes in moderate-to-vigorous physical activity (MVPA) and sedentary time (ST) among 1565 cardiovascular disease (CVD) patients using validated questionnaires at 5 weeks after lockdown initiation (i.e., baseline, April 2020) and at every 4 subsequent weeks, until July 2020. Multivariate mixed model analyses were performed to identify whether age, sex, CVD-subtype, lockdown adherence and mental health factors impacted changes in physical (in)activity. Patients were 67 (interquartile range: 60–73) years and primarily diagnosed with coronary artery disease. Time spent in MVPA was 143 min/day (95% confidence interval (CI) 137; 148) at baseline. Female sex, heart-failure, fear of COVID-19 infection and limited possibilities for physical activity were independently associated with lower levels of MVPA across time. After adjusting for confounders, overall MVPA did not change. ST was 567 (95% CI 555; 578) min/day at baseline. Lack of social contact, limited possibilities for physical activity and younger age were independently associated with higher levels of ST. After adjusting for confounders, ST progressively increased following 8 (Δ+19.7 (95% CI 0.4; 39.0)) and 12 weeks (Δ+25.2 (95% CI 5.4; 47.1) min/day) of lockdown. Despite a phased relaxation of the lockdown, CVD patients progressively increased ST and reported no change in MVPA. This highlights the need to target physical inactivity during and beyond the COVID-19 pandemic.

## 1. Introduction

To limit the spread of the severe acute respiratory syndrome coronavirus 2 (SARS-CoV-2), lockdown restrictions have been instituted worldwide. These measures have had a major impact on everyday life activities, including physical activity and sedentary behaviour in the home environment, in relation to commuting and during leisure time activities [1,2]. The majority of cross-sectional studies performed in the general population reported an acute decrease in physical activity and a simultaneous rise in sedentary time after the introduction of lockdown restrictions [3]. 

Cardiovascular disease (CVD) patients are not only at increased risk for severe COVID-19 complications [4], but given the deleterious health effects of an inactive lifestyle, they are also more prone to CVD progression and have a higher risk of recurrent cardiovascular events and all-cause or cardiovascular mortality [5,6,7]. Whilst CVD patients already demonstrate lower physical activity patterns compared to their healthy peers [8], their physical activity further deteriorated after the introduction of COVID-19 lockdown restrictions [9]. A study of 26 heart failure patients described a decrease of 16.2% in daily step count during the first 3 weeks of lockdown [10]. However, it is currently unknown whether these acute effects on physical activity and time spent sedentary persist following a prolonged lockdown period. Withdrawing restrictions made it possible to re-engage in (supervised) physical activity and exercise training. Nevertheless, no previous study prospectively evaluated physical activity patterns across the various phases of the COVID-19 lockdown, while it is clinically important to assess whether an inactive lifestyle may become more prevalent in CVD patients [11,12]. Identifying factors associated with detrimental changes in physical activity and sedentary behaviour could enable a targeted approach in CVD patients to prevent physical inactivity associated health risks during and after the COVID-19 lockdown. 

In this study, we prospectively evaluated changes in physical activity and sedentary behaviour among chronic CVD patients during the first-wave COVID-19 lockdown in the Netherlands and aimed to identify patient and disease characteristics associated with physical inactivity. Given the gradual relaxation of restrictions across the study period (April–July 2020), but also due to seasonal changes in physical activity patterns, we hypothesized an increase in physical activity levels and attenuation of sedentary behaviour in CVD patients during the first-wave COVID-19 lockdown in the Netherlands.

## 2. Materials and Methods

### 2.1. Study Population

We invited CVD patients who were included in an ongoing multicenter study (*n* = 2178) [13] via e-mail to participate in this prospective cohort study assessing longitudinal changes in self-reported physical activity and sedentary behaviour during the COVID-19 lockdown. Patients were recruited within four hospitals in the Netherlands (i.e., Radboud university medical center, Rijnstate Hospital, Jeroen Bosch Hospital and Isala Clinic) in collaboration with the Dutch Heart Foundation. Inclusion criteria were CVD diagnosis and/or referral to cardiac rehabilitation between 2015 and 2018. The study conforms to the ethical guidelines of the 1975 Declaration of Helsinki and was approved by the local medical ethics committee of the Radboud university medical center, Nijmegen, The Netherlands (ref. 2020–6414). All participants provided informed consent. 

### 2.2. Study Procedures

**Figure 1:**
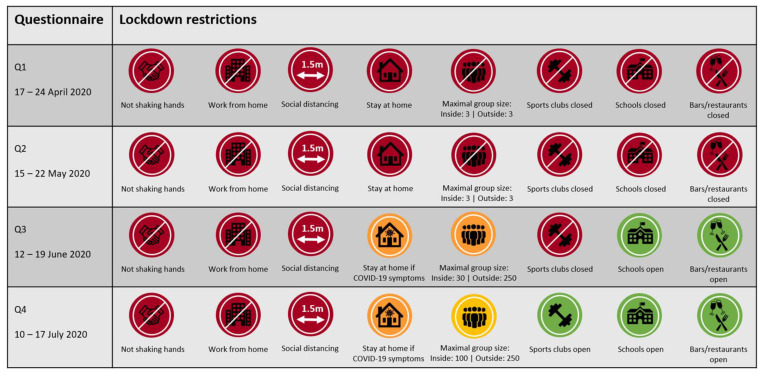
Overview of lockdown restrictions in the Netherlands applicable at the time of the assessment of physical activity and sedentary behaviour during the first-wave of COVID-19.

Physical activity and sedentary behaviour were assessed using online questionnaires at four different timepoints. Baseline data were collected between 17 and 24 April 2020 (Q1), i.e., 36 days after initiation of the COVID-19 lockdown in the Netherlands. Data on follow-up measurements were collected every subsequent four weeks, i.e., 15–22 May 2020 (Q2); 12–19 June 2020 (Q3) and 10–17 July 2020 (Q4). Dutch lockdown restrictions varied within the study period and for each timepoint of data collection, the applicable measures are summarized in Figure 1. 

Daily amount of moderate-to-vigorous intensity physical activity (MVPA) was assessed by the Short Questionnaire to Assess Health-Enhancing Physical Activity (SQUASH) [14]. We calculated the daily time spent on physical activities with a Metabolic Equivalent of Task (MET) value of at least 3.0, according to the Adult Compendium of Physical Activities [15]. Daily time spent performing MVPA was determined in four different settings: work, transportation, household and leisure time. Leisure time activities consisted of walking, cycling, doing odd jobs (e.g., doing repairs, washing the car, painting), gardening and performing exercise. 

Sedentary behaviour was assessed using the Sedentary Behaviour Questionnaire (SBQ) [16], determining time spent sedentary in nine distinct everyday life situations (i.e., watching television, using a tablet, computer or game console, eating and drinking, listening to music, talking on the phone, reading, doing arts and crafts, deskwork and transportation by car, bus or train). These items were stratified into work, transportation and leisure time sitting. Total daily sedentary time was calculated by multiplying weekdays estimates by 5 and weekend days estimates by 2 and subsequently dividing the sum by 7.

We determined age, sex and CVD subtype at baseline. Adherence to the lockdown restrictions was assessed by asking to what extent patients adhered to the applicable COVID-19 lockdown restrictions at the four different timepoints. We used a 10-point Likert scale with 0 representing no adherence and 10 representing complete adherence. Additionally, we repeatedly evaluated the extent to which patients were impeded in their everyday life by a lack of social contact; fear of COVID-19 infection; possibilities for physical activity and stress due to financial consequences of the COVID-19 pandemic. The extent of experiencing these limitations was categorized into low, moderate or high. 

### 2.3. Statistical Analysis

Data were reported as number (%) for categorical variables, mean (±standard deviation) for normally distributed continuous variables and median (interquartile range) for non-normally distributed continuous variables. Changes in physical activity and time spent sedentary were analyzed with mixed models analyses using random intercepts. Time was described as categorical variable for baseline (Q1), follow-up after 4 weeks (Q2), after 8 weeks (Q3) and after 12 weeks (Q4). Furthermore, multivariate mixed model analyses were performed using forward stepwise selection to identify whether age, sex, CVD subtype, lockdown adherence and mental health factors impacted changes in physical activity and sedentary behaviour over time. This approach allowed us to take the variation within sub-populations into account and to correct for these confounding factors in the adjusted model. All statistical tests were two-sided and significance was set at *p* < 0.05. Analyses were performed with IBM SPSS Statistics-25 (IBM Corp., Armonk, NY, USA). Original individual data are available upon request.

## 3. Results

In total, 1565 patients participated in this longitudinal cohort study (response-rate: 72%). At baseline and 4, 8 and 12 weeks of follow-up, questionnaires regarding physical activity were completed by 1565; 1004; 919 and 871 CVD patients, and for sedentary behaviour by 1490; 1000; 918 and 870 CVD patients, respectively. 

### 3.1. Cohort Characteristics 

Participants were 67 (60–73) years old, mostly male (73%) and diagnosed with myocardial infarction (48%), angina pectoris (18%), heart valve disease (9%), heart failure (8%) or other CVD (17%; Table S1). These characteristics did not differ from non-participants (*n* = 609, data not shown). Adherence to lockdown restrictions was very high (9 (8–10)) at baseline, but gradually decreased across the various phases of COVID-19 lockdown (Δ −0.7 (95% confidence interval (CI) −0.8; −0.6) after 12 weeks). The extent of being impeded due to a lack of social contact was high in the majority of our patients (60%). To a lesser extent, they were impeded by fear of a COVID-19 infection (high: 46%; moderate: 18%) and limited possibilities for physical activity (high: 42%; moderate: 11%). A minority was highly limited because of stress due to financial consequences (high: 20%; moderate: 19%; Table S1). After 12 weeks of lockdown, when the sports clubs reopened again, 31% of our patients were still highly impeded by limited possibilities for physical activity. Overall, the number of patients experiencing limitations in their everyday life as a consequence of the lockdown decreased after (partly) lifting restrictions (Table S2). 

### 3.2. Changes in Physical Activity

**Figure 2:**
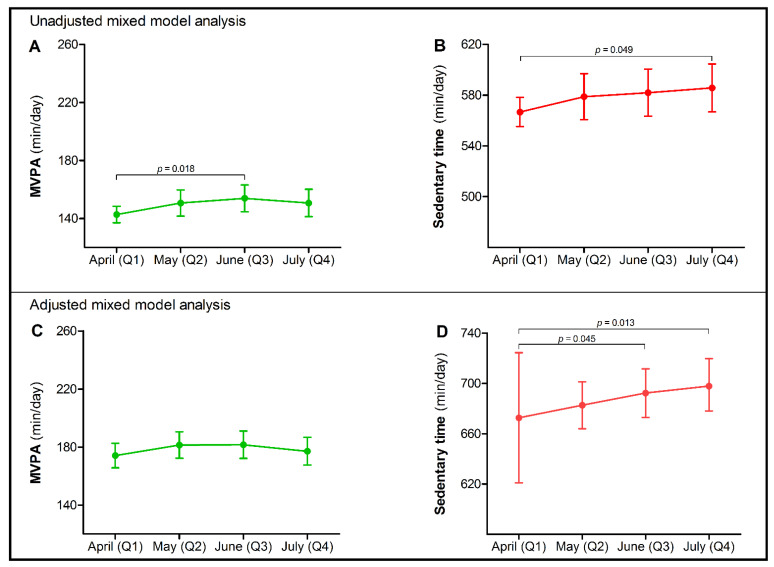
Moderate-to-vigorous physical activity (MVPA) and sedentary time with 95% confidence intervals (CIs) in Dutch chronic cardiovascular disease (CVD) patients at four different timepoints (Q1 (reference category); Q2; Q3; Q4) during the COVID-19 lockdown in 2020. In the unadjusted mixed model analysis, a small temporary increase was found in time spent performing MVPA after 8 weeks [Δ+11.2 (95% CI 1.9; 20.5) min/day] (**A**), while a time-dependent increase in sedentary time [Δ+19.0 (95% CI 0.1; 37.8) min/day] was observed after 12 weeks of lockdown (**B**). In the final multivariate mixed model analysis, adjusted for confounding factors, MVPA did not change across time (**C**), but a progressive increase in sedentary time was found among chronic CVD patients during the COVID-19 lockdown after 8 [Δ+19.7 (95% CI 0.4; 39.0) min/day] and 12 weeks [Δ+25.2 (95% CI 5.4; 47.1) min/day] (**D**).

Daily time spent in MVPA was 143 min (95% CI 137; 148) at baseline (Q1), with a minor increase (Δ+11.2 (95% CI 1.9; 20.5) min/day) at 8 weeks (Q3), but not at 4 (Q2) or 12 weeks (Q4) (Figure 2A). Changes in MVPA during the COVID-19 lockdown were not impacted by age, lockdown adherence and change in being impeded by a lack of social contact or stress due to the financial consequences of the COVID-19 pandemic. Nevertheless, MVPA levels across all time points were lower in females, heart failure patients and in those with an increasing extent of being impeded by limited possibilities for physical activity and fear of a COVID-19 infection during the lockdown (Table 1). After adjusting for these confounding factors, MVPA did not significantly change during follow-up (Figure 2C). There was no effect modification by age or sex in the final model (i.e., no significant interaction term), suggesting that changes in MVPA were similar for different age and sex groups.

**Figure 3:**
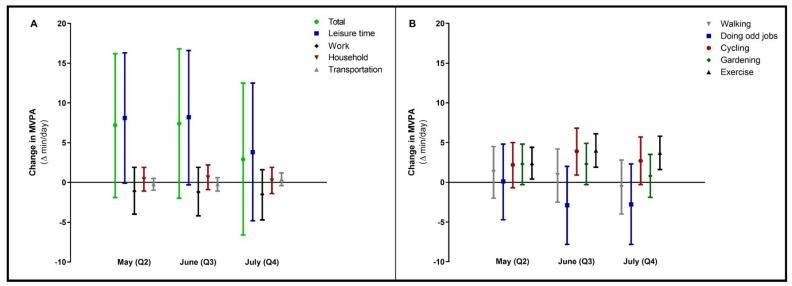
Changes in moderate-to-vigorous physical activity (MVPA, total and per different setting in panel (**A**) during the COVID-19 lockdown period. In panel (**B**), changes in the different sub settings of leisure time MVPA were specified. Data were reported as Δ (95% confidence interval) for follow-up measurements (Q2, Q3, Q4), with baseline (Q1) as reference category. Values were based on final multivariate mixed model analysis, adjusted for confounding factors.

Most of the time in MVPA was spent during leisure time activities, with 152 (95% CI 32; 272) min/day in the adjusted model at baseline (Table S3). No significant changes were found in leisure time, household, work or transportation-related MVPA during the lockdown (Figure 3A). However, within leisure time, time spent exercising significantly increased after 4, 8 and 12 weeks, compared to baseline (Q1; Figure 3B).

### 3.3. Changes in Sedentary Behaviour

Sedentary time was 567 (95% CI 555; 578) min/day at baseline and progressively increased (Δ +19.0 (95% CI 0.1; 37.8), *p* = 0.049) after 12 weeks of COVID-19 lockdown (Q4) (Figure 2B). Changes in sedentary time were not impacted by sex, CVD subtype, lockdown adherence, fear of a COVID-19 infection or stress due to financial consequences. However, daily time spent sedentary was higher in relatively younger patients and in those with an increasing extent of being impeded by a lack of social contact and limited possibilities for physical activity (Table 2). Adjusted for these confounding factors and compared to baseline (Q1), sedentary time significantly increased to 19.7 (95% CI 0.4; 39.0) min/day after 8 weeks (Q3) and to 25.2 (95% CI 5.4; 47.1) min/day after 12 weeks (Q4) (Figure 2D). There was no effect modification by age or sex in the final model.

**Figure 4:**
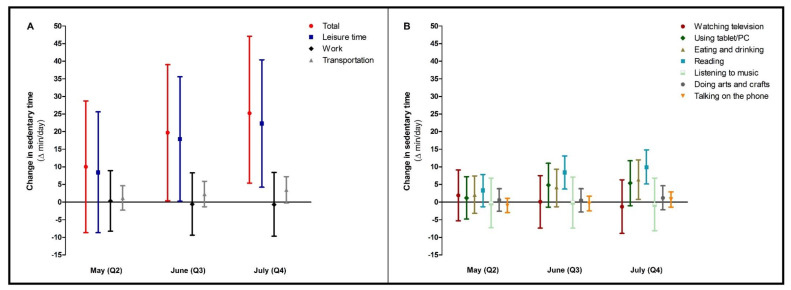
Changes in sedentary time (total and per different setting, (**A**)) during the COVID-19 lockdown period. In (**B**), changes in the different sub settings of leisure time sitting were specified. Data were reported as Δ (95% confidence interval) for follow-up measurements (Q2, Q3, Q4), with baseline (Q1) as reference category. Values were based on final multivariate mixed model analysis, adjusted for confounding factors.

Most of the time spent sedentary was work-related, with 405 (95% CI 381; 429) min/day in the adjusted model at baseline, which did not change during follow-up (Table S3). The increase in time spent sedentary was predominantly due to an increase in leisure time sitting (Figure 4A). Within leisure time, time spent eating and drinking and reading progressively increased (Figure 4B).

## 4. Discussion

The purpose of this study was to assess changes in physical activity and sedentary behaviour in CVD patients across the various phases of COVID-19 lockdown in the Netherlands. During 12 weeks of follow-up, COVID-19 lockdown restrictions were gradually lifted. Nonetheless, physical activity levels did not significantly change, while time spent sedentary progressively increased. Factors that limited engagement in physical activity were female sex, heart failure, fear of a COVID-19 infection and limited possibilities for physical activity. Fewer possibilities for physical activity, but also relatively younger age and lack of social contact, provoked sedentary behaviour. The lack of improvement in physical activity levels and further increase in sedentary behaviour, despite easing COVID-19 lockdown restrictions, is worrisome and could lead to detrimental health effects in the mid- to long-term [2,7,11].

To the best of our knowledge, this is the first prospective cohort study that assessed longitudinal changes in physical activity and sedentary time during the COVID-19 pandemic in chronic CVD patients. We found that daily time spent in MVPA did not change during follow-up, despite partial lifting of lockdown restrictions. These observations are contradictory to a previous study in the general population, showing that smartphone-based daily step counts gradually increased with prolongation of the lockdown and/or relaxing restrictions [17]. There are several possible explanations for these discrepant outcomes. First, the largest decrease in physical activity after introduction of lockdown restrictions was described in the individuals who were very active in the pre-COVID era [18,19]. Since it is known that habitual physical activity levels are lower in CVD patients compared to the general population [8,13,20], the acute impact of the lockdown on physical activity as well as changes during follow-up may be attenuated. Second, the potential benefit of reopening sports clubs to increase overall MVPA levels was limited by a concurrent decreasing trends in time spent walking and doing odd jobs in our population. Furthermore, the contribution of exercise to the total amount of MVPA was only minor. Actually, it was expected that time spent in MVPA would increase from spring to summer, because consistent patterns of seasonal influence on physical (in)activity are known to occur [21]. Nevertheless, MVPA levels did not significantly change. Another possible explanation could be the interference of anxiety or fear for COVID-19 infections, especially since CVD patients are a group vulnerable to infection [4]. This may have prevented CVD patients from (re-)engaging in physical activity and exercise training—also reflected by the large proportion of patients that still experienced limited possibilities for physical activity despite the reopening of sports clubs. This observation requires specific attention, as it suggests that the COVID-19 lockdown further aggravated the already low levels of physical activity and engagement in exercise training among CVD patients.

After 12 weeks of lockdown, sedentary time increased by 25 min/day, adjusted for age and changes in the extent of being impeded by lack of social contact and limited possibilities for physical activity. This substantial increase in sedentary behaviour was primarily due to an accumulation of time spent sitting during leisure time activities, specifically during reading, eating and drinking. These observations have important implications, since a large majority of CVD patients were already highly sedentary before the lockdown [9,13,22]. We previously reported an initial absolute increase in sedentary time of 55 min/day, directly after the introduction of COVID-19 lockdown measures in the Netherlands [9]. Extrapolating these findings, our data suggest that daily sedentary time increased by 80 min (+17%) compared to the pre-COVID era, leading to a substantially increased health risk in the short- and long term [11,23,24].

The pathways that underlie these sitting-induced health risks are still not fully understood, but probably act through multiple biological systems including vascular function, blood pressure, carbohydrate metabolism and cerebral blood flow [12]. This could explain the poorer cardiorespiratory fitness in sedentary CVD patients [25]. Furthermore, a sedentary lifestyle is strongly associated with an increased risk of atherosclerotic CVD events [26] while, in the long term, sedentary CVD patients have a higher risk of (premature) cardiovascular death [7,27]. Importantly, in the elderly population the detrimental health effects of sitting also appear to be reversible, even after adjusting for potential confounders such as smoking, hypertension, obesity and diabetes [28]. This suggests that reducing sedentary time potentially attenuates the physiological perturbations and may ultimately even reduce cardiovascular health risks [12].

The impact of the COVID-19 lockdown on physical activity and sedentary behaviour in our study were, at least partly, influenced by patient- and disease characteristics, but not by lockdown adherence or stress due to the financial consequences of the COVID-19 pandemic. The type of CVD diagnosis, sex and the extent of being impeded in daily life by limited possibilities for physical activity and fear of a COVID-19 infection impacted physical activity across time. Specifically, MVPA levels were lower in patients with heart failure compared to the other CVD groups during the COVID-19 lockdown. This is in line with previous research that described lower habitual physical activity levels in heart failure patients [29], with a further decrease during the lockdown [10]. Additionally, MVPA levels were lower in female patients and those with an increasing extent of being impeded by limited possibilities for physical activity and fear of a COVID-19 infection. During the lockdown, daily time spent sedentary was higher in relatively younger patients and in those with an increasing extent of being impeded by limited possibilities for physical activities and by a lack of social contact. Since younger CVD patients were more likely to be employed than older patients, they probably had to work from home, which could explain their higher levels of sedentary behaviour [3]. Insight into correlates of low MVPA levels and high levels of sedentary behaviour is important, as it may facilitate a targeted approach in improving activity patterns in these unprecedented times.

A large amount of chronic CVD patients reported that they were impeded in their everyday life by a lack of social contact, fear of a COVID-19 infection and limited possibilities for physical activity. With the reopening of sports clubs, patients again had full access to sports facilities and therefore the opportunity to re-engage in (supervised) physical activity. However, many patients still experienced limited possibilities for physical activity, presumably due to a persistently high level of fear of a COVID-19 infection in this vulnerable population. Possibly, loosening lockdown restrictions actually increased the risk perception in patients with regard to a COVID-19 infection. Last year, Lippi and colleagues [30] already addressed a potential deterioration in physical activity patterns as a result of the lockdown restrictions, and this unfavourable prospect was confirmed by our data. Although preventing the spread of COVID-19 still has the highest priority in most countries across the world, the health crisis will probably result in a further escalation of physical inactivity [12], which, moreover, does not seem to be automatically resolved by simply lifting lockdown restrictions. Despite the fact that the excess health risks associated with a sedentary lifestyle have been well established [26], no effective large-scale public health interventions targeting sedentary behaviour exist. The current COVID-19 pandemic accelerated the availability and implementation of eHealth initiatives in healthcare and cardiac telerehabilitation, offering scalable services to large groups of patients [31,32,33]. Remote delivery of cardiac rehabilitation now seems feasible and has the potential to increase capacity and reach diverse patient populations, also beyond the COVID-19 era [34,35]. Moreover, usage of fitness apps appears to be effective in buffering the lockdown induced pandemic of physical inactivity [36]. This fits perfectly with the need for solutions to exercise in a safe (home) environment and overcomes current barriers due to fear of infections and limited possibilities for physical activity. Several apps even facilitate performing physical activity together with peers, which hopefully will also target the lack of social contact during the lockdown. Now is the time to capitalize on these promising developments and to flatten the worldwide curve of physical inactivity with a similar urgency to combating COVID-19.

Certain limitations apply to our study, including its observational design, which cannot confirm causality, but only describes statically significant changes in physical activity patterns during different phases of the COVID-19 lockdown and independent associations between observed changes and patient characteristics. Unfortunately, data on follow-up measures were not completed in all participants. We limited the impact of missing data due to use of mixed model analyses, but acknowledge that selection bias could not be completely ruled out. Additionally, we specifically focused on the Dutch situation and our findings may not be directly extrapolated to other countries with different types of lockdowns or with cultural differences. Furthermore, self-reported physical activity seems relatively high in our cohort, but this is mainly driven by time spent doing odd jobs and walking, while time spent during exercise was only minor. The use of subjective measurements could have led to over- and underestimation of physical activity and sedentary behaviour, respectively. Nevertheless, we performed longitudinal measurements and since the used questionnaires have good reproducibility [37], our study design allowed us to validly examine changes in physical activity and time spent sedentary across the COVID-19 lockdown. An important advantage of subjective assessment is the ability to assess domain-specific changes in physical activity (e.g., exercise; cycling) and sedentary behaviour (e.g., reading; eating and drinking). Since we have studied changes in physical activity patterns directly after initiation of lockdown restrictions, we cannot draw any conclusions about longer-term effects. Especially now it appears that short-term lockdowns will be more frequently needed to contain the spread of COVID-19, our observation highlights the need for further studies using objective measurements to prospectively follow-up CVD patients and healthy controls in relation to the current, but also future, lockdown measures.

## 5. Conclusions

Despite the gradual relaxation of lockdown restrictions across the study period, physical activity levels did not improve, while sedentary time progressively increased in chronic CVD patients during 12 weeks of COVID-19 lockdown. Female sex, relatively younger age, heart failure diagnosis and an increasing extent of being impeded by limited possibilities for physical activity and by a lack of social contact were associated with physical inactivity. Since deterioration in physical activity patterns is strongly associated with medium to long-term detrimental health effects, targeting physical inactivity should be a top priority in order to limit the collateral damage of COVID-19 lockdown restrictions. Our findings indicate that simply lifting lockdown measures does not automatically recover physical activity patterns in CVD patients, highlighting the need to (1) frequently monitor activity levels, (2) raise awareness concerning the health risks of a physically inactive lifestyle and (3) to develop (digital) solutions to effectively promote physical activity in these patients.

## Figures and Tables

**Table 1:** Final multivariate mixed model for changes in moderate-to-vigorous physical activity (MVPA) during the first-wave COVID-19 lockdown period, adjusted for confounding factors. Data were reported as estimate (95% confidence interval (CI)).

			MVPA (min/day) Estimate (95% CI)	*p*
**Questionnaire timepoint**				
	Q1—April (ref)		0	
	Q2—May		7.2 (−1.9; 16.2)	0.12
	Q3—June		7.4 (−2.0; 16.8)	0.12
	Q4—July		2.9 (−6.6; 12.5)	0.55
**Sex**				
	Male (ref)		0	
	Female		−41.4 (−49.2; −33.7)	<0.001
**CVD subtype**				
	Myocardial infarction (ref)		0	
	Angina pectoris		4.8 (−4.4; 14.1)	0.31
	Heart valve disease		−1.2 (−14.1; 11.4)	0.85
	Heart failure		−23.0 (−36.5; −9.5)	0.001
	Other *		−0.9 (−10.5; 8.7)	0.85
**Extent of being impeded by:**				
	**Fear of a COVID-19 infection**			
		Low (ref)	0	
		Moderate	−7.4 (−17.3; 2.4)	0.14
		High	−16.9 (−24.7; −9.1)	<0.001
	**Limited possibilities for physical activity**			
		Low (ref)	0	
		Moderate	−23.3 (−34.8; −11.9)	<0.001
		High	−18.2 (−25.9; −10.6)	<0.001

* Other was defined as heart rhythm disorders, congenital heart disease, stroke and peripheral artery disease. CVD, cardiovascular disease; ref, reference category.

**Table 2:** Final multivariate mixed model for changes in sedentary time during the first-wave COVID-19 lockdown period, adjusted for confounding factors. Data were reported as estimate (95% confidence interval (CI)).

			Sedentary Time (min/Day) Estimate (95% CI)	*p*
**Questionnaire timepoint**				
	Q1—April (ref)		0	
	Q2—May		10.0 (−8.7; 28.7)	0.29
	Q3—June		19.7 (0.4; 39.0)	0.045
	Q4—July		25.2 (5.4; 47.1)	0.013
**Age** (years) *			−2.1 (−2.8; −1.3)	<0.001
**Extent of being impeded by:**				
	**Lack of social contact**			
		Low (ref)	0	
		Moderate	13.0 (−11.3; 37.4)	0.30
		High	23.3 (6.6; 40.1)	0.006
	**Limited possibilities for physical activity**			
		Low (ref)	0	
		Moderate	15.1 (−8.9; 37.4)	0.22
		High	41.4 (24.9; 57.8)	<0.001

* Per 1 year increase. Ref, reference category.

## Data Availability

Original individual data are available upon request.

## Supplementary Materials

The following are available online at https://www.mdpi.com/article/10.3390/ijerph182211929/s1, Table S1: Baseline characteristics, Table S2: Extent of being impeded by mental health factors during follow-up, Table S3: Changes in moderate-to-vigorous physical activity (MVPA) and sedentary time during the first-wave COVID-19 lockdown period.
