# Supplementary material for: Changes in Physical Activity and Sedentary Behaviour in Cardiovascular Disease Patients during the COVID-19 Lockdown
Source: Int J Environ Res Public Health. 2021 Nov 13;18(22):11929. doi: 10.3390/ijerph182211929 (PMC8623455; doi:10.3390/ijerph182211929)
Supplement: Supplementary file 1 [file ijerph-18-11929-s001.zip › ijerph-1431611-supplementary.pdf]

Supplementary material s –

Changes in Physical Activity and Sedentary Behaviour in Cardiovascular Disease Patients during the COVID-19 Lockdown.

Supplemental Tables

Table S1. Baseline characteristics.

Baseline characteristics		*n* = 1,565	Missing values
**Age (years)**		67 [60, 73]	0 (0%)
**Sex (male)**		1144 (73%)	0 (0%)
**CVD subtype**			0 (0%)
Myocardial infarction		754 (48%)	
Angina pectoris		284 (18%)	
Heart valve disease		139 (9%)	
Heart failure		119 (8%)	
Other*		269 (17%)	
**Lockdown adherence (0-10)**		9 [8, 10]	4 (0.3%)
**Extent of being impeded by:**			
Lack of social contact			68 (4%)
	Low	438 (29%)	
	Moderate	168 (11%)	
	High	891 (60%)	
Fear of a COVID-19 infection			18 (1%)
	Low	560 (36%)	
	Moderate	273 (18%)	
	High	714 (46%)	
Limited possibilities for physical activity			22 (1%)
	Low	728 (47%)	
	Moderate	174 (11%)	
	High	641 (42%)	
Stress due to financial consequences of COVID-19			2 (0.1%)
	Low	955 (61%)	
	Moderate	300 (19%)	
	High	308 (20%)	

*Other was defined as heart rhythm disorders, congenital heart disease, stroke and peripheral artery disease. Data were reported as n (%) or median [interquartile range]. COVID-19, coronavirus disease 2019; CVD, cardiovascular disease.

Table S2. Extent of being impeded by mental health factors during follow-up.

Extent of being impeded in by:	May (Q2)	June (Q3)	July (Q4)
**Lack of social contact**			
Low	315 (33%)	350 (40%)	398 (48%)
Moderate	88 (9%)	111 (13%)	109 (13%)
High	553 (58%)	419 (48%)	328 (39%)
**Fear of a COVID-19 infection**			
Low	405 (41%)	425 (47%)	417 (49%)
Moderate	157 (16%)	147 (16%)	161 (19%)
High	430 (43%)	332 (37%)	282 (33%)
**Limited possibilities for physical activity**			
Low	475 (49%)	480 (54%)	474 (57%)
Moderate	96 (10%)	98 (11%)	100 (12%)
High	396 (41%)	311 (35%)	262 (31%)
**Stress due to financial consequences of COVID-19**			
Low	655 (66%)	626 (69%)	620 (72%)
Moderate	167 (17%)	152 (17%)	129 (15%)
High	174 (18%)	136 (15%)	118 (14%)

Table S3. Changes in moderate-to-vigorous physical activity (MVPA) and sedentary time during the first-wave COVID-19 lockdown period*.

	**April (Q1)**	**May (Q2)**		**June (Q3)**		**July (Q4)**	
**Moderate-vigorous physical activity**	Estimate (95% CI)	Δ (95% CI)	*P*	Δ (95% CI)	*P*	Δ (95% CI)	*P*
**Total MVPA (min/day)**	174.2 (165.7; 182.6)	+7.2 (-1.9; 16.2)	0.12	+7.4 (-2.0; 16.8)	0.12	+2.9 (-6.6; 12.5)	0.55
Leisure-time	152.1 (32.3; 271.9)	+8.1 (-0.1; 16.3)	0.05	+8.2 (-0.3; 16.6)	0.06	+3.8 (-4.8; 12.5)	0.38
Doing odd jobs	51.9 (47.4; 56.4)	0.1 (-4.7; 4.8)	0.98	-2.9 (-7.8; 2.0)	0.25	-2.8 (-7.8; 2.3)	0.28
Walking	38.7 (35.7; 41.8)	+1.3 (-2.0; 4.5)	0.44	+0.9 (-2.5; 4.2)	0.60	-0.6 (-4.0; 2.8)	0.73
Cycling	30.2 (-136.4; 196.8)	+2.2 (-0.7; 5.0)	0.14	+3.9 (0.9; 6.8)	0.011	+2.7 (-0.3; 5.7)	0.08
Gardening	19.2 (16.9; 21.6)	+2.3 (-0.3; 4.8)	0.09	+2.3 (-0.3; 4.9)	0.09	+0.8 (-1.9; 3.5)	0.56
Exercise	12.1 (-58.0; 82.1)	+2.4 (0.4; 4.4)	0.021	+4.0 (1.9; 6.1)	<0.001	+3.7 (1.6; 5.8)	0.001
Household	9.4 (-0.4; 19.3)	+0.4 (-1.1; 1.9)	0.59	+0.6 (-0.9; 2.2)	0.43	+0.2 (-1.4; 1.9)	0.76
Work	10.8 (-53.5; 75.1)	-1.1 (-4.0; 1.9)	0.48	-1.2 (-4.2; 1.9)	0.44	-1.5 (-4.7; 1.6)	0.33
Transportation	1.9 (1.1; 2.6)	-0.2 (-1.0; 0.5)	0.54	-0.2 (-1.0; 0.6)	0.64	+0.4 (-0.4; 1.2)	0.35
**Sedentary time**	Estimate (95% CI)	Δ (95% CI)	*P*	Δ (95% CI)	*P*	Δ (95% CI)	*P*
**Total sedentary time (min/day)**	672.7 (621.0; 724.4)	+10.0 (-8.7; 28.7)	0.29	+19.7 (0.4; 39.0)	0.045	+25.2 (5.4; 47.1)	0.013
Leisure-time	216.6 (168.4; 264.9)	+8.4 (-8.7; 25.6)	0.33	+17.9 (0.26; 35.6)	0.047	+22.3 (4.2; 40.4)	0.016
Watching television	117.2 (19.5; 215.0)	+1.9 (-5.3; 9.1)	0.61	+0.1 (-7.4; 7.5)	0.99	-1.3 (-8.9; 6.3)	0.74
Using tablet/PC	76.5 (59.9; 93.1)	+1.2 (-4.8; 7.2)	0.70	+4.8 (-1.4; 11.0)	0.13	+5.4 (-1.0; 11.7)	0.10
Eating and drinking	42.7 (-73.9; 159.2)	+2.1 (-3.2; 7.4)	0.44	+4.2 (-1.3; 9.6)	0.14	+6.4 (0.8; 12.0)	0.026
Reading	-66.1 (-167.8; 35.6)	+3.3 (-1.3; 7.8)	0.16	+8.4 (3.7; 13.1)	<0.001	+9.9 (5.1; 14.8)	<0.001
Listening to music	20.2 (-179.8; 220.2)	-0.3 (-7.3; 6.8)	0.94	-0.1 (-7.4; 7.1)	0.97	-0.7 (-8.1; 6.8)	0.86
Doing arts and crafts	18.2 (8.3; 28.1)	+0.6 (-2.6; 3.8)	0.70	0.5 (-2.8; 3.8)	0.77	1.2 (-2.2; 4.6)	0.48
Talking on the phone	8.9 (-8.2; 26.0)	-1.0 (-3.0; 1.1)	0.35	-0.4 (-2.5; 1.7)	0.68	+0.8 (-1.4; 2.9)	0.49
Deskwork	404.9 (381.2; 428.7)	+0.3 (-8.3; 8.9)	0.94	-0.6 (-9.4; 8.3)	0.90	-0.7 (-9.7; 8.4)	0.88
Transportation by car, bus or train	51.3 (-22.1; 124.6)	+1.2 (-2.3; 4.6)	0.51	+2.3 (-1.3; 5.9)	0.21	+3.5 (-0.2; 7.2)	0.06

*Data were reported as estimate (95% confidence interval (CI)) for baseline (Q1) measurements and Δ (95% CI) for follow-up measurements (Q2, Q3, Q4). Values were based on final multivariate mixed model analysis, adjusted for confounding factors with baseline (Q1) as reference category.
